# Generation of Induced Pluripotent Stem Cells from CD34+ Cells across Blood Drawn from Multiple Donors with Non-Integrating Episomal Vectors

**DOI:** 10.1371/journal.pone.0027956

**Published:** 2011-11-22

**Authors:** Amanda A. Mack, Stacie Kroboth, Deepika Rajesh, Wen Bo Wang

**Affiliations:** Cellular Dynamics International, Inc., Madison, Wisconsin, United States of America; University of South Florida, United States of America

## Abstract

The methodology to create induced pluripotent stem cells (iPSCs) affords the opportunity to generate cells specific to the individual providing the host tissue. However, existing methods of reprogramming as well as the types of source tissue have significant limitations that preclude the ability to generate iPSCs in a scalable manner from a readily available tissue source. We present the first study whereby iPSCs are derived in parallel from multiple donors using episomal, non-integrating, oriP/EBNA1-based plasmids from freshly drawn blood. Specifically, successful reprogramming was demonstrated from a single vial of blood or less using cells expressing the early lineage marker CD34 as well as from unpurified peripheral blood mononuclear cells. From these experiments, we also show that proliferation and cell identity play a role in the number of iPSCs per input cell number. Resulting iPSCs were further characterized and deemed free of transfected DNA, integrated transgene DNA, and lack detectable gene rearrangements such as those within the immunoglobulin heavy chain and T cell receptor loci of more differentiated cell types. Furthermore, additional improvements were made to incorporate completely defined media and matrices in an effort to facilitate a scalable transition for the production of clinic-grade iPSCs.

## Introduction

Fibroblasts have been a predominate source material for the development of the process to generate induced pluripotent stem cells (iPSCs) given their ability to expand, endure for multiple passages in culture, and receptiveness to efficient infection by viruses expressing a combination of transcription factors for reprogramming [1]–[6]. However, fibroblasts from skin biopsies require invasive surgical procedures, are labor intensive and isolating a sufficient number for reprogramming takes time. In addition, the ability to generate iPSCs from skin appears inversely correlated with the age of the donor likely due to increasing exposure to external mutagens [7]. There is value, therefore, in alternative tissue sources to generate iPSCs that minimize the risk for additional mutation, involve less invasive procedures, and are amenable to industrialization to increase availability across an extensive population range.

A blood draw is an ideal starting point to generate donor-specific iPSCs because it is minimally invasive and established procedures are already in place for acquisition and handling [8]. Lymphocytes comprise a large fraction of the peripheral blood mononuclear cell (PBMC) population but pose at least two potential limitations. First, they are subject to intrinsic DNA rearrangements such as those that occur in B and T cells at the V, D, and J gene segments as well as T cell receptor (TCR) loci to generate a diverse repertoire of antigen-specific surface immunoglobulins. These rearrangements are subsequently perpetuated in iPSCs generated from them and their impact on iPSC function is currently unknown [9], [10]. Second, some research has indicated that host cell types may influence functional properties of iPSCs [11], [12]. For example, while embryonic stem (ES) cells and progenitor cells derived from bone marrow successfully differentiate into B cells, iPSCs derived from B cells have demonstrated resistance to this ability [13], [14], [15]. Therefore, choosing an early lineage cell type that lacks DNA rearrangements alleviates the potential risk of reduced ability to differentiate.

Somatic cells that are characteristically more progenitor-like with respect to the expression of early lineage markers, such as CD34, appear more susceptible to reprogramming, and they too can be isolated from blood [16]. For example, Haase and colleagues successfully isolated and reprogrammed early progenitor cells isolated from cord blood. The ability to reprogram cells from peripheral blood, however, expands the range of host cells available for reprogramming especially when acquisition of cord blood-derived material is not an option. However, the amount of CD34+ cells represents less than 0.1% of the population of PBMCs thus limiting the amount of source material available for reprogramming. To generate enough starting material to perform reprogramming trials, Loh and colleagues relied on patients treated with granulocyte-colony stimulating factor (G-CSF) to expand the number of CD34+ cells in circulating peripheral blood and ultimately generated iPSCs from these cells [17]. The acquisition of blood that does not require donors to receive these agents would be more desirable to avoid the negative side effects associated with them [18]. Studies have also shown that cells from mobilized blood demonstrate functional differences when compared with cells from non-mobilized samples indicating changes to properties intrinsic to the cell [19]. For example, epigenetic and genetic anomalies (i.e. aneuploidy) have been detected in cells derived from patients mobilized with G-CSF [20], [21]. These observations increase the likelihood that similar genetic modifications would carry over into iPSCs generated from mobilized CD34+ cells and potentially impact their function.

The method to generate iPSCs from cord and mobilized peripheral blood has predominately relied on viral-based methods to introduce reprogramming factors [16], [17]. Resulting clones thus have transgenes integrated into their genomes that may alter the function of iPSCs, increase the risk of cancer, and hinder their potential for clinical application. Improvements to reprogramming methods have been made to eliminate integrated transgenes including a recent study examining the reprogramming potential of blood-derived cells using a previously described episomal, oriP/EBNA1-based transfection method [2], [22], [23]. We capitalized on the oriP/EBNA1-method but made modifications to accommodate the reprogramming of CD34+ cells derived from actual vials of blood collected across multiple donors. The oriP/EBNA1-based vectors contribute to the replication and retention of plasmids during each cell division long enough for reprogramming to occur and are lost over time resulting in cells free of transfected DNA and integrated transgenes [24], [25]. Importantly, reprogramming can be achieved through a single transfection. Herein we demonstrate the ability to generate iPSCs from a single vial of blood or less using an improved process of reprogramming that incorporates fully defined conditions to generate iPSCs free of gene rearrangements and transgene elements.

## Results

### Hematopoietic progenitor cells from non-mobilized, peripheral blood are expandable

**Figure 1:**
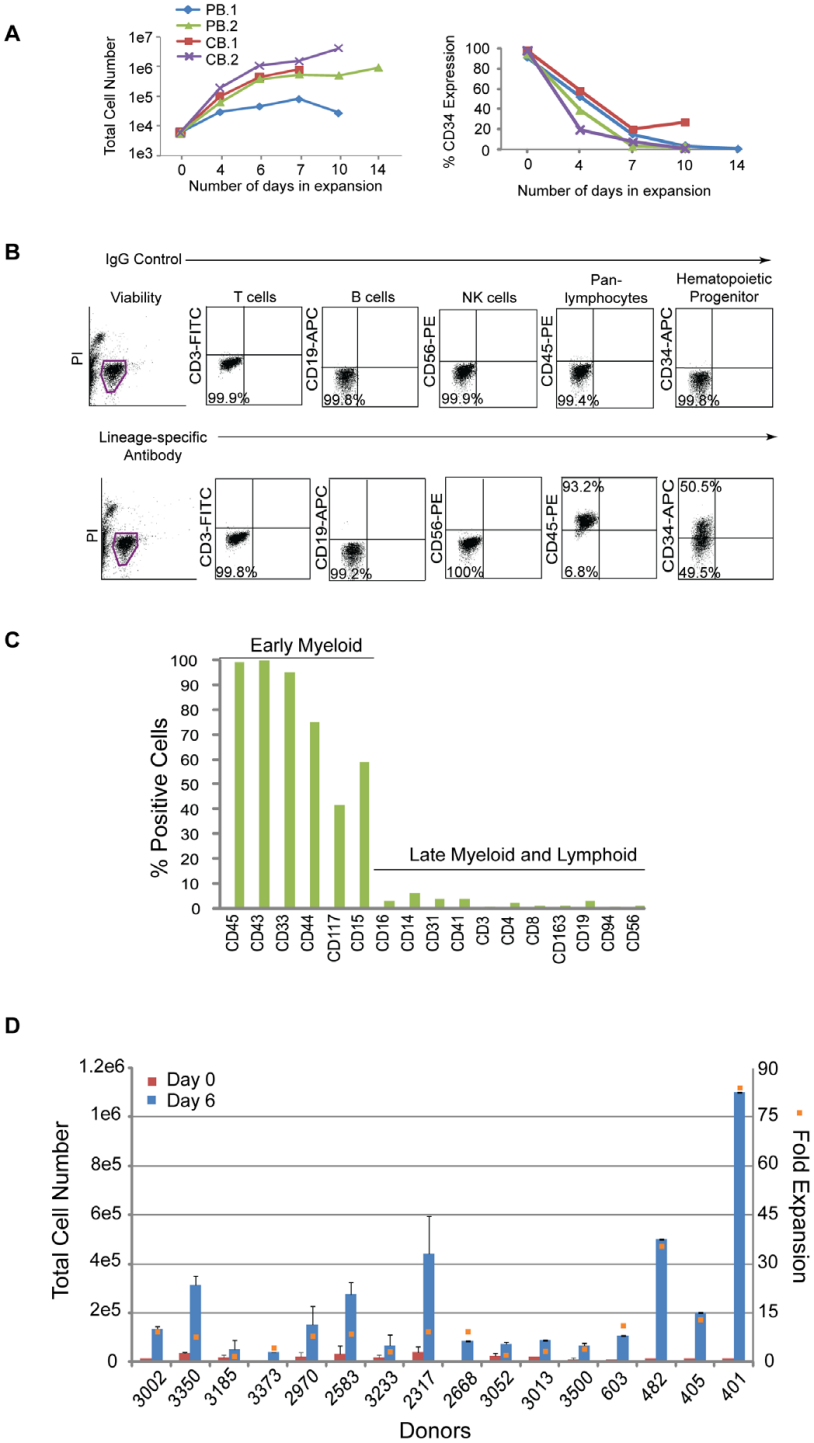
Hematopoietic cells enriched for CD34 expression are expandable. A. Graphs depict the expansion of purified cells from either two peripheral (PB.1 and PB.2) or cord (CB.1 or CB.2) blood donors over time (lefthand panel) along with the percentages of the total population that are CD34+ (righthand panel). B. Representative profile of a purified population of cells after 6 days of expansion by flow cytometry on cells isolated from Donor 3002. Flow cytometry plots from control staining using IgG antibodies (upper plots) are compared to plots with antibodies specific to lineage markers (lower plots). C. The graph represents an extended analysis by flow cytometry of the characteristic profile of PB.2 cells after 10 days of expansion. The % positive indicates the fraction of the population expressing the cell surface markers on the x-axis. D. The total number of CD34+ cells across 16 different donors was assessed beginning at 0 and 6 days of expansion (left side y-axis). The fold expansion (right side y-axis, orange squares) was determined by dividing the total number of cells at day 6 divided by the number of cells at day 0 after purification. The average percent of CD34 expression across all 16 donors was 48+/−19%.

Hematopoietic progenitor cells expressing CD34 represent a small fraction of the population and limit the number of cells available for reprogramming; therefore, a modified formulation of a media used to expand cord blood cells was tested on CD34+ cells isolated from peripheral blood [26]. CD34+ cells from two non-mobilized, peripheral (PB.1 and PB.2) blood donors were tested in comparison to CD34+ cells from two cord blood donors (CB.1 and CB.2). Cells were placed into untreated, 24-well culture plates at 1.2×10^4^ per ml of expansion media (StemSpan basal medium; 300 ng/ml each of SCF, Flt3, TPO; 100 ng/ml IL-6; 10 ng.ml IL-3) and fed 3 to 4 days later. The total number of cells from peripheral blood expanded 170-fold and 680-fold from cord blood after 10 to 14 days in culture (Figure 1A, left hand panel). The percentage of CD34+ cells in the peripheral and cord blood samples peaked in less than 1 week (CB data not shown; Figure 1A, right hand panel). The expression profile of expanding populations was determined by flow cytometry for all donors examined herein (Figure 1B). Phenotypic analysis of the resulting peripheral blood culture PB.2 after 10 days of expansion revealed predominately early progenitor cells expressing CD43, CD45, CD33, CD44, CD15, and CD117 and marginal levels of T (CD3, CD4, CD8), NK (CD56, CD94), B (CD19), macrophage (CD163), megakaryocyte (CD41), and monocyte (CD14) cells (Figure 1C).

These expansion conditions were then applied to cells acquired from a single vial of blood collected from multiple donors. PBMCs were isolated, frozen down immediately, or directly purified for CD34+ cells and seeded for expansion. On average, 1×10^7^ PBMCs were recovered per 8 ml vial of blood and yielded approximately 2×10^4^ cells after purification for CD34-expressing cells (data not shown). Although the magnitude of expansion was variable, cells from all of the donors demonstrated expansion ranging from 3 to 83-fold after 6 days in culture and approximately 48+/−19% of that population expressed CD34 (Figure 1D).

### Optimizing the generation of iPSCs with small molecules and a defined matrix

**Figure 2:**
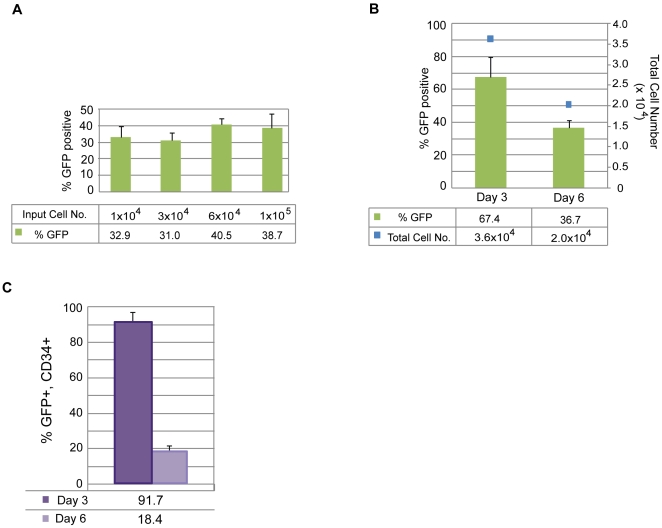
Identifying optimal transfection conditions for CD34+ cells. A. PBMCs (donor GG) were isolated and purified for CD34-expression and expanded for 6 days. A range of cell numbers were transfected with a control, oriP/EBNA1-based plasmid expressing GFP. Transfection efficiency was determined by calculating the percentage of viable cells expressing GFP detectable by flow cytometry (n = 6). B. PBMCs (donor A2389) were isolated, purified for CD34-expression and expanded for 3 or 6 days. 6×10^4^ to 1×10^5^ cells were transfected with the control, GFP-expressing plasmid. The graph depicts the percent of the total population that is GFP-positive along with the absolute number of total cells (n = 3). C. The graph represents the fraction of cells in B that co-express GFP and CD34 when transfected at 3 or 6 days of expansion (n = 3).

The total number of purified cells isolated from a single, 8 ml vial of blood can be as little as 2×10^4^ CD34+ cells; therefore, a range of CD34+ cell numbers was tested to determine transfection efficiency from low cell numbers. The efficiency of transfection was determined by transfecting an oriP/EBNA1-containing plasmid encoding GFP into expanded CD34+ cells and assessing them by flow cytometry. Viability was determined by identifying the fraction of viable cells that did not stain positively for trypan blue the day after transfection divided by the total number of input cells. Viability was approximately 30% when 1×10^4^ to 1×10^5^ input cells were used for transfection (data not shown). Cell numbers at 1×10^4^ and 3×10^4^ resulted in an efficiency of 30% and was 40% when using 6×10^4^ and 1×10^5^ cells (Figure 2A). Cells expanded for only 3 days demonstrated a two-fold increase in transfection efficiency. Over 90% of those cells also co-expressed GFP and CD34 while only 18% of the cells transfected after 6 days of expansion co-expressed both markers (Figure 2B, C). These results support the notion that the conditions selected for this protocol favor the transfection of CD34+ cells present in the population.

**Figure 3:**
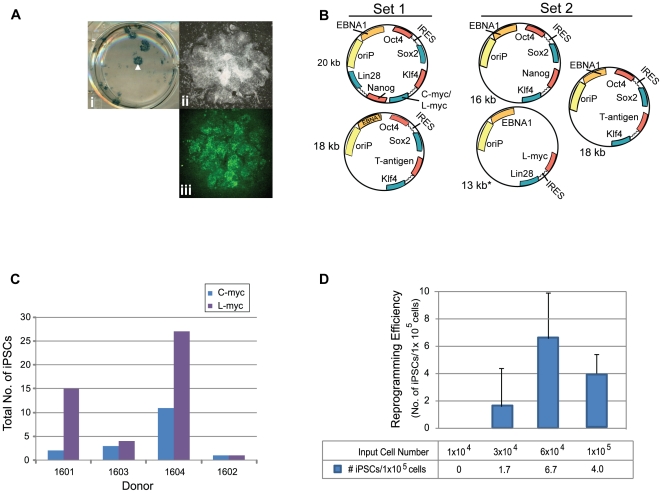
Plasmid transfections to optimize reprogramming efficiency. A. Representative reprogramming trial from freshly drawn blood (donor 3002) using combination plasmid Set 2 for transfection. A single well is shown from a 6-well plate that contains colonies staining positively for AP activity (i). The white arrowhead highlights the colony magnified in panel ii that also stained positively for Tra-1-81 expression (green), panel iii. B. Schematic of the plasmid sets successfully used for reprogramming trials. Set 1 contains a combination of two plasmids for transfection whereby a 20 kb plasmid that either contains C- or L-myc is depicted. Set 2 includes a three plasmid combination for transfection. C. CD34+ cells purified from four different donors were expanded for 6 days and transfected using the plasmid combination that expresses either Set 1 or Set 2 plasmid sets to compare the total number of resulting iPSCs. D. Reprogramming trials were performed using plasmid Set 2 to transfect a range of cell numbers expanded for 6 days (donor GG, n = 6).

We anticipated variability in reprogramming efficiency given the differences already observed across donors for other cell types tested and with other methods of reprogramming. Therefore, we optimized the matrix, media, and plasmid combinations used for reprogramming. Firstly, a common source of variation occurs when MEFs or matrigel are used because both are undefined, cumbersome to prepare, and vary from lot-to-lot. Therefore, we established a defined matrix by testing a variety of commercially available possibilities. Recombinant protein fragments containing the active domains of human fibronectin (RetroNectin) or vitronectin consistently supported iPSC formation the best among those tested. Second, the efficiency of colony formation on RetroNectin-coated plates improved significantly when used in combination with StemSpan SFEM media, N2, B27, and a cocktail of small molecules that included PD0325901, CHIR99021, A-83-01, and HA-100 (Figure 3A). These molecules have been described previously as inhibitors of MEK, GSK3β, TGFβ, and ROCK pathways, respectively [27], [28]. Patches of adherent cells appeared within one week and became a positive indicator for progression into iPSCs since hematopoietic cells are typically cultured in suspension. The following week many of the colonies exhibited overt characteristics typical of an iPSC and stained positively for the common pluripotency markers Tra-1-81 and alkaline phosphatase (AP) (Figure 3A). The borders of the colonies were compact and the nucleoli more visible when cultures were transitioned to defined, TeSR2 media without small molecules 1.5 to 2 weeks following transfection. Thirdly, episomal oriP/EBNA1-based plasmids were used to deliver Oct4, Sox2, Klf4, C-myc, Nanog, Lin28, and SV40 Large T-antigen as previously described (Set 1; Figure 3B) [2]. Different combinations of reprogramming plasmids were also tested to determine whether a boost in reprogramming efficiency was possible. Based on previous reports indicating the benefit of L-myc in reprogramming trials, we modified plasmid combination Set 1 and substituted L-myc in place of C-myc (Set 2, Figure 3B) [29], [30]. While an improvement was not observed when C-myc was substituted for L-myc in the combination of plasmids represented in Set 1 (data not shown), an equal to or two-fold improvement was observed with plasmid Set 2 expressing L-myc (Figure 3C). Optimizing a range of input cell numbers for transfection also revealed more consistent generation of iPSCs when transfecting greater than 5×10^4^ cells (Figure 3D).

### Optimization of iPSCs generated from CD34+ cells isolated from fresh, whole blood across multiple donors

The next step was to confirm iPSCs could be generated from actual vials of human blood, ensure cell numbers optimized for expansion and transfection are applicable across multiple donors, and determine whether the starting volume of blood can be minimized. Colonies emerging during reprogramming were scored positive by their ability to express Tra1-81 and exhibit a classic embryonic stem (ES) cell-like morphology. After colonies were picked from the reprogramming cultures, a subset of them were further characterized to confirm their pluripotency. Reprogramming efficiency was calculated in two ways 1) the number of iPSCs divided by the total volume of blood collected from each donor and 2) the total number of iPSCs divided by the number of cells for transfection multiplied by 1×10^5^ cells. The first calculation incorporates the whole process beginning from the blood collection to the generation of an iPSC. The second calculation removes the variability incurred during the isolation of PBMCs, purification, and expansion and focuses on the number of iPSCs generated per number of cells placed into transfection.

We tested blood collected across donors spanning a range of ethnicities, ages, and genders to confirm iPSCs could be generated from CD34+ cells purified from fresh blood draws (Table 1). Six of these donors provided up to 55 ml of blood, and PBMCs from them were either isolated and used directly for purification, expansion, and reprogramming or frozen down after isolation. iPSCs were successfully generated from all six donors regardless of whether they were from fresh or frozen cells despite the lower efficiency of reprogramming, less than 1 iPSC per ml of blood (Table 2). Next, smaller volumes of blood representative of a single vial were obtained from six different donors to test parameters established in earlier experiments such as the number of cells for transfection and plasmid combinations. The cell numbers used for transfection from donors 3052, 3233, and 3373 ranged from 2×10^4^ to 4×10^4^ which fall below the minimum, 5×10^4^ cells, established with our optimization studies. These experiments resulted in less than one iPSC per ml of blood (Table 2). Also, transfections with cells from donors 2583, 2970, and 3185 represent early trials performed with plasmid Set 1 expressing C-myc before Set 2 plasmids expressing L-myc were fully optimized which may have resulted in more iPSCs.

**Table 1:** Diversity across the set of donors used for reprogramming trials to generate iPSCs.

Donor ID	Ethnicity	Gender	Age
GG	Caucasian	M	39
A2389	Caucasian	M	47
401	Caucasian	M	40–60
405	Caucasian	M	40–60
482	Caucasian	M	40–60
603	Caucasian	M	40–60
2317	Caucasian	M	29
2341	Hispanic	M	30
2364	Caucasian	M	29
2369	Caucasian	M	27
2447	Caucasian	F	50
2583	African A.	M	51
2668	African A.	M	34
2726	African A.	M	38
2849	African A.	M	29
2939	Asian	F	32
2970	Asian	M	29
3002	Asian	F	25
3013	Caucasian	M	48
3052	Caucasian	M	21
3096	African A.	M	32
3098	Caucasian	F	22
3185	African A.	M	42
3233	Asian	M	22
3264	African A.	F	33
3323	Caucasian	F	35
3350	Caucasian	M	47
3373	African A.	F	47
3389	Caucasian	M	22
3415	African A.	F	32
3428	Caucasian	F	24
3436	African/Native A.	F	39
3447	Hispanic	F	34
3500	Caucasian	M	39
3663	Caucasian	F	50
3665	African A.	F	25
3675	Hispanic	F	42

The ethnicity, gender, and age of all of the donors providing non-mobilized, whole blood for the reprogramming trials. Blood was collected from all of the donors, except GG and A2389, using standard blood drawing procedures. Donors GG and A2389 underwent leukapheresis to provide PBMCs. African A. indicates African American; M, male; F, female. A range rather than a specific age was provided for donors 401, 405, 482, and 603.

**Table 2:** Optimizing reprogramming from a range of blood volumes across multiple donors.

Volume of blood (ml)	Donor	iPSCs/ml of blood	iPSCs/1×105 cells	Set 1(N)	Set 2(N)
**>16**	**3428**	0.1	1.9	1	1
**>16**	**2939**	0.3	3.8	3	1
**>16**	**3323**	0.4	1.4	1	
**>16**	**2369**	0.9	2.9	1	1
**>16**	**2364**	0.1	0.7	1*	
**>16**	**3415**	1.2	6.6	1	1
**8**	**3052**	0.8	8.9		2*
**8**	**3233**	0.4	9.4		2*
**8**	**3373**	0.1	2.4	1	
**8**	**2583**	0.5	1.7	4*	
**8**	**2970**	0.3	1.6	5*	
**8**	**3185**	0.5	4.5	1*	

The table represents reprogramming efficiencies following optimization trials that began with a set of donors providing different volumes of blood. “iPSCs per ml blood” represents the number of total iPSCs as determined by their ability to stain positively for Tra1-81 expression and exhibit characteristic, ES-like morphology divided by the volume of blood processed from the donor. “iPSCs per 1×10^5^” cells represents the number of total iPSCs divided by the total number of cells used for each transfection after 6 days of expansion multiplied by 1×10^5^ cells. Set 1 refers to plasmid combination Set 1 expressing C-myc and Set 2 refers to the triple plasmid combination set using expressing L-myc. Asterisks indicate the generation of iPSCs from CD34+ cells isolated fresh from blood while all other trials were performed from CD34+ cells isolated from PBMCs that were frozen down immediately after isolation from blood. N indicates the number of independent reprogramming trials performed for each donor. Reprogramming efficiencies were averaged where N>1.

The next step was to then extend the insights acquired from these donors and verify the robustness of our protocol against ten new donors. The average number of iPSCs per ml of blood and per 1×10^5^ cells across these donors indeed improved after incorporating experience with handling and the testing performed on the earlier donor samples (Table 3). Furthermore, these experiments were extended to test even smaller volumes of blood from a subset of the same donors in Table 3. CD34+ cells purified from approximately 4 ml of blood were sufficient to generate iPSCs from all six donors tested, and CD34+ cells from approximately 2 ml of blood from four out of six of these donors generated iPSCs (Table 4). These results demonstrate that iPSCs can be generated from CD34+ isolated from tractable volumes of blood using this non-intergrating and feeder-free method of reprogramming.

**Table 3:** Improved reprogramming across multiple donors from a single vial of blood.

Donor	iPSCs/ml of blood	iPSCs/1×105 cells	Set 1(N)	Set 2(N)
**3002**	4.5	24.7	2*	
**3350**	2	3.1	2*	
**2317**	7.4	59.3		4
**2668**	6	55.2		1
**3013**	3.8	50		1
**3500**	4.6	55.2		2
**603**	10.6	77.3		1
**482**	12	19.2		1
**405**	1.3	5		1
**401**	10.5	8.4		1

The table represents reprogramming trials beginning from a single vial of blood performed across a set of donors independent from Table 2. Set 1 refers to the two plasmid combination expressing C-myc and Set 2 involves the 3 plasmid combination set expressing L-myc. Asterisks indicate the generation of iPSCs from CD34+ cells isolated fresh from blood while all other trials were performed from CD34+ cells isolated from PBMCs that were frozen down immediately after isolation from blood. N indicates the number of single vial reprogramming trials performed for each donor. Reprogramming efficiencies were averaged where N>1.

**Table 4:** The efficiency of reprogramming CD34+ cells beginning from 4 ml of blood or less.

Donor	Half Vial			Quarter Vial		
	iPSCs/ml blood	iPSCs/1×105 cells	N	iPSCs/ml blood	iPSCs/1×105 cells	N
3500*	1.3	8.3	2	10.0	178.5	2
**3052**	2.7	31.5	2	0	0	1
**401**	10.0	6.6	1	4.5	4.9	2
**405**	3.0	8.8	1	1.8	13.5	2
**603**	0.5	4.1	1	0.3	10.0	1
**482**	3.0	37.3	1	0	0	1

Single, 8 ml vials of blood were fractionated from a subset of the donors from Table 3 to represent half or one-quarter of a whole vial. PBMCs were isolated and CD34+ cells purified from them for use in the reprogramming method described here. Asterisks indicate the generation of iPSCs from CD34+ cells isolated fresh from blood while all other trials were performed from CD34+ cells isolated from PBMCs that were frozen down immediately after isolation. Plasmid combination Set 2 expressing L-myc was used for transfection on cells from each of these donors. N indicates the number of independent reprogramming trials performed. Reprogramming efficiencies were averaged where N>1.

### iPSCs derived from peripheral blood are pluripotent and free of transgene elements

**Figure 4:**
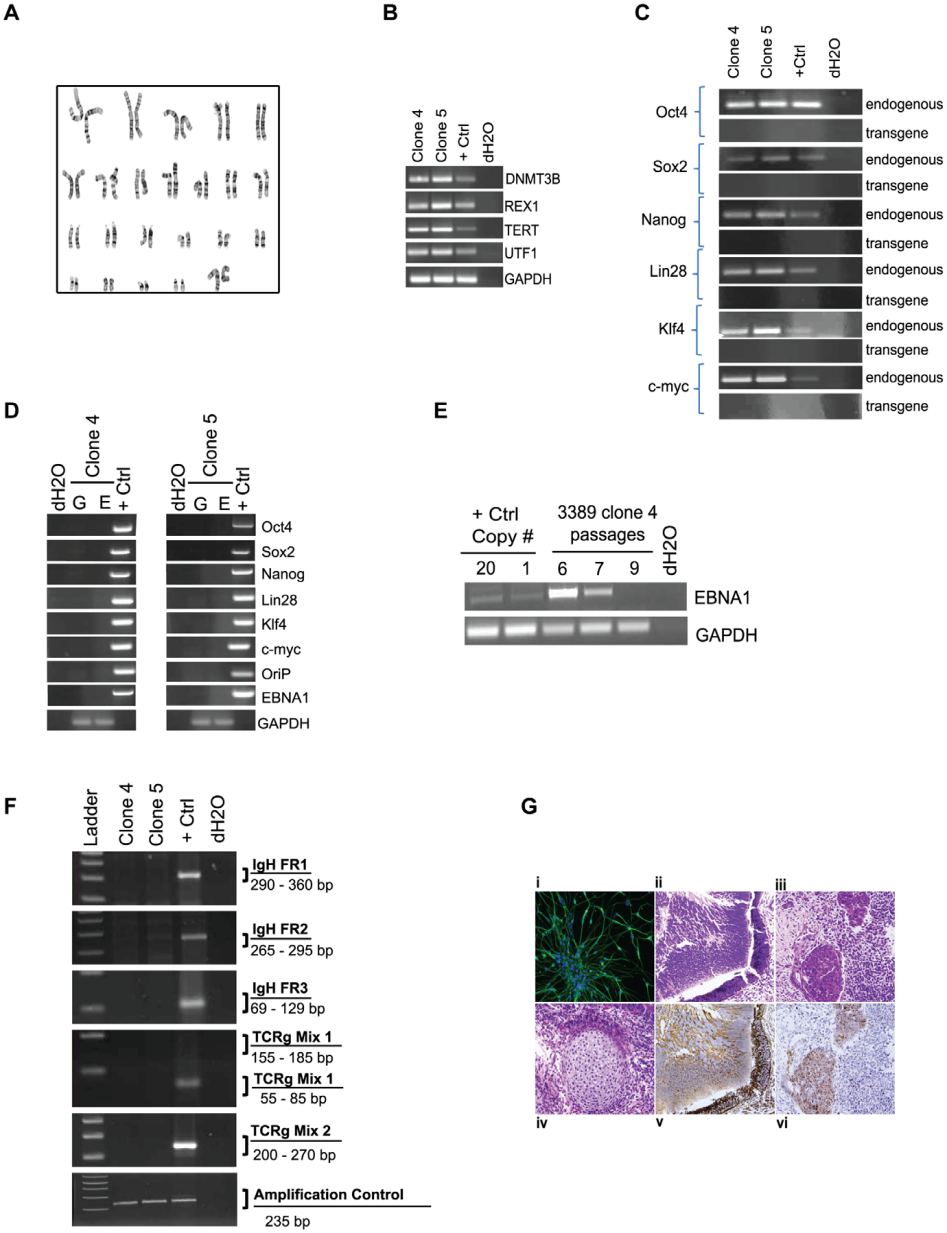
Characterization of iPSCs derived from CD34+ blood cells. A subset of iPSC clones were characterized for pluripotency. The experiments demonstrated in this figure provide representative examples of the types of results observed for characterization studies using iPS clones 4 and/or 5 derived from donors 2939 and 3389. A. Cytogenetic analysis on G-banded metaphase cells from iPS clone 4 exhibiting a normal karyotype. B and C. RT-PCR confirms the endogenous expression of classic pluripotency genes and the absence of expression from transgenes. A standard in-house iPS line served as the positive control k. D. Clones were deemed free of episomal (E) DNA and genomic integration (G) by PCR. E. PCR was used to track the loss of oriP/EBNA1-based plasmids at multiple passages using primers that amplify EBNA1. A control plasmid at 1 and 20 copies per genome was used to establish the sensitivity of the PCR at 1 copy per 3,000 cells. F. PCR screen using primers specific for the joining region and all three of the conserved framework regions (FR1, FR2 and FR3) to amplify immunoglobulin heavy chain (IgH) gene rearrangements and two assays with primers specific to the T cell receptor (TCR) gamma gene rearrangement. G. Representative image of donor 2939 clone 5 differentiated in vitro into neurons (i). Clone 5 also demonstrated differentiation into all three germ layers: ii) epithelium iii) endoderm iv) mesoderm v) ectoderm vi) endoderm from teratomas formed when iPSCs were injected into immunodeficient, SCID mice.

**Figure 5:**
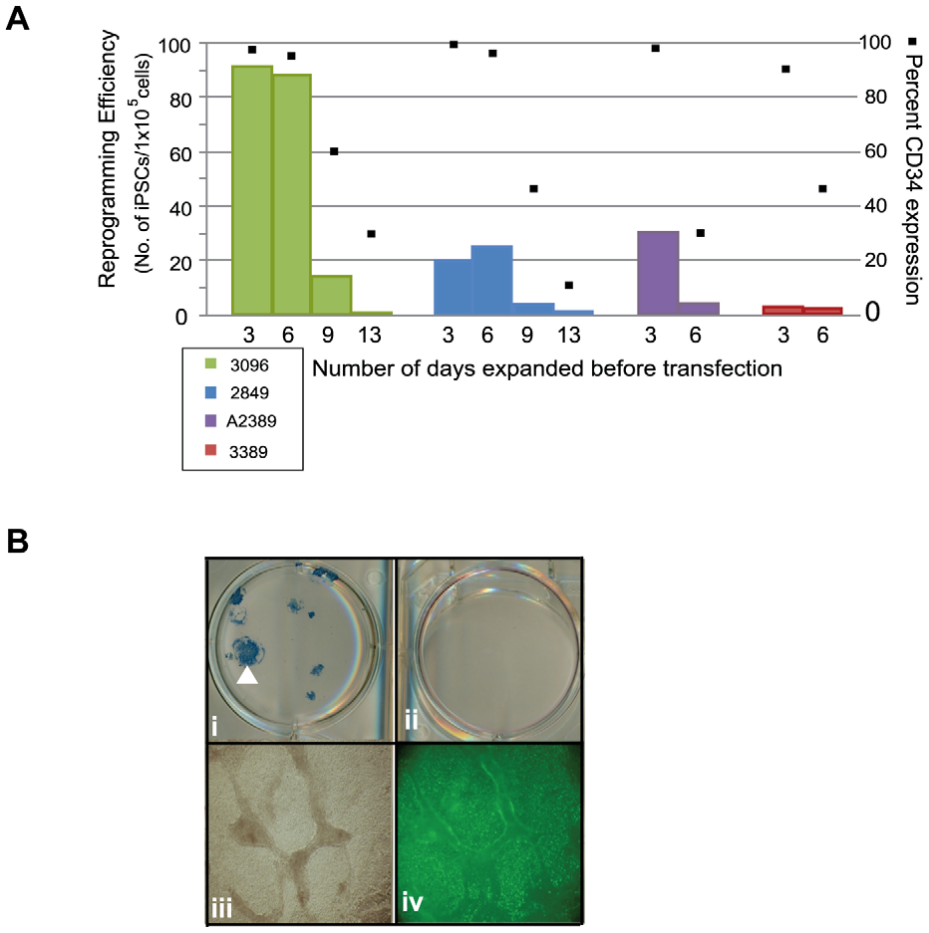
The presence of CD34+ cells correlates with reprogramming efficiency. A. CD34+ cells from four different blood donors were expanded for 3, 6, 9, or 13 days. A large volume of blood was collected from donors 3096, 2849, and 3389 to ensure sufficient cell numbers to perform these studies. Expanding CD34+ cells using plasmid DNA combination Set 2. The efficiency of reprogramming was calculated as the total number of iPSCs exhibiting morphological features characteristic of an ES cell and an ability to stain positively for Tra-1-81 divided by the total number of cells used for transfection. Black Squares depict the percentage of the population expressing CD34 at the indicated days of expansion. B. Representative reprogramming trial whereby both the positive (i) and negative (ii) fraction following purification were used for reprogramming. Panel (i) shows one well of a 6-well plate that contains successfully reprogrammed colonies from donor 2939 based on their ability to demonstrate AP activity. The CD34-depleted fraction from donor 2939 was unable to form colonies as indicated by the lack of AP staining when performed in parallel with the purified population panel, ii. Panels iii and iv magnify the colony in panel (i) marked by a white arrowhead and demonstrates expression of Tra-1-81 (green), panel iv.

Multiple iPSCs from each of the donors that were reprogrammed from Tables 2, 3, and 4 were selected for further characterization to confirm their pluripotency. The clones exhibited a normal karyotype, were positive for Tra-1-81 and SSEA-4 expression by flow cytometry as well as endogenous genes DNMT3B, REX1, TERT, UTF1, Oct4, Sox2, Nanog, Lin28, Klf4, and C-myc (Table 5, Figure 4A–C). Clones did not exhibit integrated transgene or episomal elements and loss of episomal DNA occurred, on average, within 7–10 passages (Table 5, Figure 4D,E). A PCR screen did not reveal rearrangements pertaining to immunoglobulin heavy chain (IgH) or a subset of T cell receptor (TCR) gene segments (Table 5, Figure 4F). The lack of rearrangements supports the notion that the protocol selectively favors the production of iPSCs from hematopoietic progenitors rather than more differentiated cell types. When used for *in vitro* directed differentiation at passage 15, donor 2939 iPSC clones 4 and 5, which have lost episomal plasmids, were competent to form neurons (Figure 5G). Furthermore, five iPS clones from three different donors also formed teratomas after injection into immunodeficient (SCID) mice (Figure 4G). Interestingly, the presence of residual episomal plasmids did not appear to hinder the ability to form teratomas since clone 6 from donor 2970 did not lose transfected plasmids until passage 18, well after injection into mice for teratoma studies.

**Table 5:** Characterization of insert-free iPS clones derived from fresh blood.

			Passage Number							
Donor	Clone No.	Tra1-81 live stain	IgH/TCR	Loss of oriP	Endogenous Expression	Integration Free	Karyotype	Teratomas	% Tra1-81+	% SSEA-4+
**3002**	1	+	7	n/a	7	7	6	7	85.6	81.2
	4	+	7	n/a	7	7	6	7	90.2	81.0
**2939**	4	+	11	9	18	18	11, 13	9	82.0	84.0
	5	+	12	10	18	18	8, 11	10	86.3	86.3
**2970**	6	+	18	18	24	24	3, 25	8	84.1	90.8
**3389**	4	+	10	9	10	10	12			
	5	+	10	9	10	10	12			
	6	+	10	9	10	10	12			
	1	+	n/a	9						
	2	+	n/a	9						
	3	+	n/a	9						
**3233**	4.2-2	+	n/a	9						
	1.1-1	+	10	9						
	1.4-4	+	10	9						
	4.2-1	+	10	9						
**3185**	3.3	+	10	7						
**3052**	5	+	3	n/a						
	6	+	3	n/a						
**3500**	11	+	3	n/a						
	12	+	3	n/a						

This table represents the passage numbers at which further characterization of a subset of the iPSCs generated across different blood donors were examined. All clones positively stained for the pluripotency marker Tra-1-81. The clones were free of detectable IgH and TCR gene rearrangements (IgH/TCR). A subset of these clones were also screened by PCR using multiple primer sets for loss of the transfected, oriP/EBNA1 DNA including those for EBNA1. The numbers indicate the passage where amplified products are no longer detected (Loss of oriP). The expression of endogenous genes was verified by RT-PCR and included DNMT3B, REX1, TERT, UTF1, Oct4, Sox2, Nanog, Lin28, Klf4, and C-myc (Endogenous Expression). Clones were deemed free of integrated DNA by PCR using primers specific for segments of the transfected DNA encoding Oct4, Sox2, Nanog, Lin28, Klf4, and C-myc (Integration Free). Genetic integrity of the clones was confirmed by cytogenetic analysis at one or more passages where indicated. Teratomas were generated by injecting iPSCs at the indicated passage number into immunodeficient mice. Clones were also assessed by flow cytometry using antibodies specific for cell surface expression of both Tra-1-81 and S SEA-4. n/a indicates tests not performed with the indicated clone. All clones satisfied our minimal criteria for an iPSC by exhibiting a classic ES-like morphology and stained positively for Tra-1-81 and AP.

### Reprogramming efficiency correlates with the amount of CD34-expression

**Figure 6:**
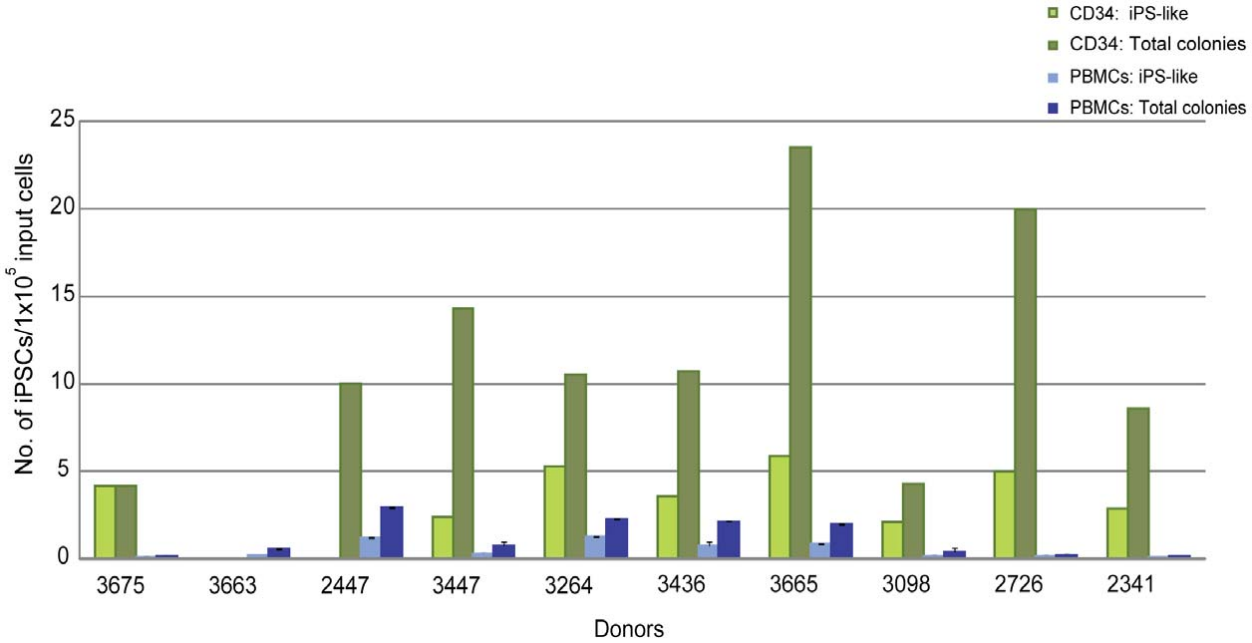
Comparing the efficiency of reprogramming between PBMCs and CD34+ cells. PBMCs and CD34+ cells were isolated from single tubes of blood provided from 10 different donors. The efficiency of reprogramming following transfection with DNA Combination Set 2 was determined for each donor and each method. “Total colonies” refers to all iPS colonies derived from either CD34+ or PBMC populations that stain positively for Tra1-60 and “iPS-like” colonies are those that stain positively for Tra1-60, exhibit clear iPS morphology, and are large enough to pick for expansion. Input cells refer to the number of CD34+ cells or PBMCs were used for transfection. The efficiencies across all donors from both methods were compared using the Wilcoxon signed rank test (two-sided), p = 0.007.

The isolation of CD34+ cells from PBMCs creates an additional step in our process and others have demonstrated successful reprogramming directly from PBMCs without the need for purification [22]. Therefore, several experiments to determine whether a correlation exists between CD34+ cells and reprogramming efficiency were performed. First, the expanding CD34+ populations were screened by flow cytometry for characterization prior to transfection. T, B, and NK cells were undetectable after 3 and 6 days of expansion demonstrated by their lack of CD3, 19, and 56 expression, respectively. The percentage of CD34 expression during expansion ranged from 30 to 100%, thus increasing the likelihood of reprogramming more of an early lineage cell type (n = 9, data not shown). Second, samples were taken from the purified populations during expansion at different timepoints as they lost CD34 expression to determine their receptiveness to reprogramming. A decrease in reprogramming efficiency was observed in correlation with decreasing percentages of CD34 expression across populations of cells from four independent donors (Figure 5A). For example, donor 3096 exhibited only 1 iPSC per 1×10^5^ input cells when beginning from cells expanded for 13 days (31% CD34+) compared to 91.5 iPSCs per 1×10^5^ cells following 3 days when levels of CD34 expression were much higher, 98% (Figure 5B). Third, populations depleted of CD34+ cells were tested for their ability to reprogram in parallel with their CD34+ cell counterparts. These CD34-depleted populations were not receptive to reprogramming as the CD34+ cells even when the same media and transfection conditions were used (n = 3, Figure 5B). Finally, a side-by-side comparison of reprogramming efficiency was performed between unpurified PBMCs and CD34+ cells isolated from 10 different donors. A medium described previously for the expansion of erythroblasts and for successful reprogramming studies was used to ensure media would not be a limiting factor for reprogramming the PBMCs in our protocol [22], [31]. Reprogramming trials beginning from either PBMCs or CD34+ cells were launched in parallel using their respective media for expansion. The efficiency of reprogramming was approximately 2 to 8 fold higher when beginning with cells purified for CD34 expression in 9 out of the 10 donors compared to those from PBMCs (p = 0.007; Figure 6). A significant fraction of the PBMC population was comprised of lymphocytes (∼79+/−14% CD3/CD19) at the time of transfection (data not shown); therefore, PCR was performed to screen for potential IgH and TCR gene rearrangements to determine whether both protocols promoted the generation of iPSCs free of gene rearrangements. Interestingly, screened clones from either cell type were free of IgH and TCR gene rearrangements indicating that both protocols favor the reprogramming of early progenitor cells. The lower efficiency of reprogramming from the PBMC population may reflect the dilution of early progenitor blood cells by a predominately lymphocytic population.

### Generation of iPSCs from CD34+ cells using completely defined reagents

**Figure 7:**
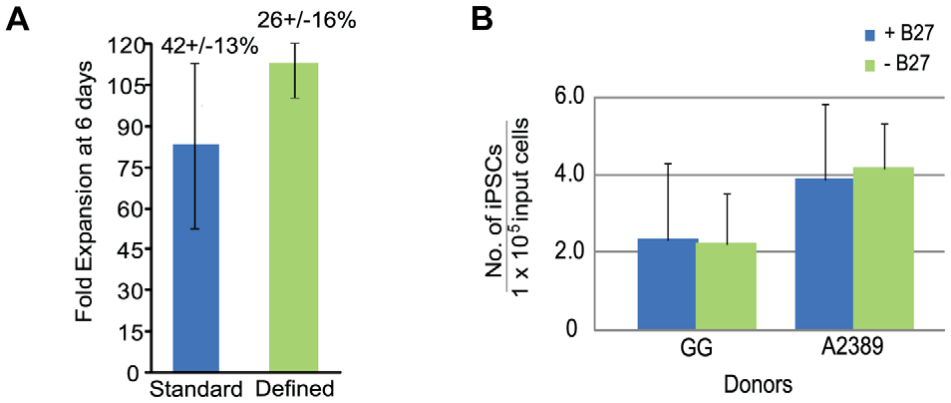
The generation of iPSCs from blood using completely defined conditions. A. Fold expansion of CD34+ cells pooled from multiple blood donors in standard (n = 13) and completely defined conditions (n = 2) after 6 days of expansion. Fold expansion was calculated from the total number of cells at day 6 divided by the number of cells the day after purification. Percentages indicate the fraction of cells expressing CD34 in the total population as assessed by flow cytometry. B. Reprogramming trials were performed on CD34+ cells obtained by leukapheresis from donors GG and A2389 with and without the B27 supplement.

Additional reprogramming trials were performed using completely defined conditions to enable the production of clinic-grade iPSCs. A large pool of CD34+ cells mixed from multiple donors was used for multiple tests and resulted in a successful expansion of 113+/−11 fold in defined media compared to 83+/−32 fold for cells in standard conditions after 6 days of expansion (Figure 7A). Despite the 30-fold difference between the two conditions, the absolute number of CD34+ cells is similar between the two populations when multiplied by the percentage of the population expressing CD34 by flow cytometry. For example, 42+/−13% of the population expanded in standard conditions expressed CD34 and 26+/−16% expressed CD34 using completely defined conditions (Figure 7A). There were no detectable CD3+, CD19+, or CD56+ cells after 6 days in culture consistent with our earlier expansion trials (data not shown). The media used for reprogramming is completely defined with the exception of the supplement B27 which contains bovine serum albumin (BSA). However reprogramming was still achieved in the presence or absence of B27 (Figure 7B). These improvements coupled with a defined matrix enables the production of iPSCs in a completely defined process.

## Discussion

CD34+ cells possess characteristics that make them an ideal blood cell to reprogram: they are readily identified, highly receptive to reprogramming, and free of gene rearrangements characteristic of more differentiated cell types. However, their low numbers in circulating blood have made them a less desirable cell type for reprogramming because large volumes of blood were predicted to be required for the generation of iPSCs. We describe a method to generate insert-free iPSCs from CD34+ cells beginning from a single vial of blood or less. In some cases, the number of CD34+ cells that expanded exceeded that required for a single transfection after 6 days in culture making it possible to transfect after only 3 days of expansion, shorter than the amount of time Chou and colleagues used to reprogram unpurified PBMCs [22].

Proliferation contributes to reprogramming efficiency; therefore, choosing a culture medium that promotes proliferation will effectively promote reprogramming. Data presented in our manuscript supports this assertion because all four donor populations examined were almost 100% positive for CD34 expression after 3 days in culture, but they were not equally receptive to reprogramming (Figure 5A). Cells from donor 3096 demonstrated the highest fold expansion following purification as well as the highest efficiency of reprogramming. However, we go on to demonstrate that proliferation is not the only contributor to efficient reprogramming. In our experiments, the purity of the cell population diminishes over time following purification, but the cells within the culture continue to expand despite low CD34 expression. Figure 1C shows that this expanding population 10 days following purification consists primarily of early lineage progenitor cells. This result indicates that our medium has the capacity to stimulate the proliferation of non-T and non-B cells that have never been or are no longer CD34+. If proliferation were the primary force driving the efficiency of reprogramming, then it would be expected that proliferating non-T/non-B cells within our population would be equally receptive to reprogramming regardless of their time in culture. Our results are contrary to this hypothesis, however, because the efficiency of reprogramming decreases as the magnitude of CD34 expression decreases. This is observed across four independent donor cell populations and is consistent with dependence of reprogramming on CD34 expression (see Figure 5A). These results, taken together, support our hypothesis that both proliferation and cell identity contribute to the efficiency of reprogramming.

We have also outlined a protocol that begins to systematically address some of the challenges in the generation of clinical-grade iPSCs in an effort to advance their use from research lab to clinic. iPSCs must not only be manufactured consistently from a tractable tissue source but also satisfy safety requirements. The core of these requirements includes the use of completely defined culture conditions and a standardized reprogramming method that results in the removal of the potentially oncogenic transgenes employed to reprogram the cells. The starting point of the protocol is the actual patient sample, a single vial of blood. The ability to begin from frozen rather than fresh starting material allows flexibility to launch multiple reprogramming trials in parallel. We demonstrate that either CD34+ cells or PBMCs may be used as the source population for reprogramming. The iPSCs generated by this method are free of transfected DNA as well as B and T cell gene rearrangements. Several challenges remain, however, before routine production of clinical-grade iPSCs can be completely performed. First, there is considerable variation in the efficiency of reprogramming from donor to donor. Some of this variation is likely due to the inherent differences among the donors, but a careful examination of external sources of variation at each step from blood to iPSCs may well reveal areas in addition to those we have uncovered that can be better controlled. For example, we demonstrate potential in the ability to produce iPSCs using completely defined reagents to minimize variation. Second, an automated method for screening and selecting iPSCs during reprogramming would facilitate high throughput production of iPSCs. Third, a robust production protocol must also include a method for the rapid screening of iPSCs to identify those that both lack potentially harmful mutations and are readily differentiated into various cell types. In sum, the generation of iPSCs using a standardized process beginning from early progenitor cells isolated from routine blood draws minimizes this variation and is a good starting point to provide a more comparable baseline for analysis. We present the first steps towards a standardized process to make the generation of clinical-grade iPSCs a reality.

## Materials and Methods

### Ethics Statement

All human primary cells were generated in vitro from tissue samples from human donors with appropriate written informed consent given to the commercial providers.

All animal work was conducted according to relevant national and international guidelines under the approval of the Cellular Dynamics International Animal Care and Use Committee. As a private company, our animal facility does not provide a permit number or approval ID since mouse is not a protected species.

### Processing whole blood samples

Peripheral (PB.1 and PB.2) and cord (CB.1 and CB.2) blood-derived CD34+ cells were obtained from AllCells (Emeryville, CA USA). Blood collections were performed at AllCells and Meriter Laboratories (Madison, WI USA) using standard, 8 ml Vacutainer Cell Processing Tubes (both sodium citrate and sodium heparin-based tubes are acceptable; BD Biosciences; Franklin Lakes, NJ USA). Appropriate documentation for informed consent was completed prior to blood collection (Meriter Laboratories). Vacutainers were processed within 24 hours of collection. Briefly, the PBMC-containing upper phase was collected and washed with ice-cold PBS (Invitrogen; Carlsbad, CA USA). Cells were either frozen down or used directly for purification with the CD34 MicroBead Kit (Miltenyi; Auburn, CA USA) and used according to the manufacturer's protocol. Some samples were treated with Histopaque (Sigma Aldrich; St. Louis, MO USA) to minimize the number of red blood cells (RBCs) and centrifuged at 2000 rpm for 20 minutes without braking. The interface containing the PBMCs was removed if samples were treated with histopaque, cells washed again with chilled PBS, centrifuged at 600× g for 15 minutes and either frozen down with CryoStor10 (StemCell Technologies; Vancouver, BC Canada) or used directly for purification. CD34+ cell expansion media: StemSpan SFEM (StemCell Technologies), Flt3, SCF, TPO each at a final concentration of 300 ng/ml, IL-6 (100 ng/ml) and IL-3 (10 ng/ml) (Peprotech; Rocky Hill, NJ USA), supplemented with DNaseI (final concentration at 20 U/ml), and 1× Antibiotic-antimycotic (Invitrogen) for overnight recovery. Defined expansion media: serum-free StemSpan H3000 (StemCell Technologies), animal-free IL-6 (R&D Systems Minneapolis, MN USA), and recombinant human IL-3, TPO, Flt3, and SCF (Peprotech) at the same concentrations listed above. PBMC expansion media: StemSpan SFEM, ExCyte Medium Supplement (Millipore; Billerica, MA), Glutamax (Invitrogen), SCF (250 ng/ml), IL-3 (20 ng/ml), Erythropoietin (2 U/ml; Prospec; Rehovot, Israel), IGF-1 (40 ng/ml; Prospec), and Dexamethasone (1 µM; Fisher; Waltham, MA). PBMCs were resuspended at 1×10^6^ cells/ml for expansion.

### Flow cytometry

Cell surface staining of hematopoietic cells was performed with CD45-PE, CD34-APC, CD19-APC and CD56-PE (BD Biosciences) and CD3-PE (eBioscience; San Diego, CA USA) antibodies. iPSCs were processed directly for antibody staining for the presence of Tra-1-81 (Stemgent; Cambridge, MA USA) and SSEA-4 (BD Pharmingen; San Diego, CA USA). Propidium Iodide (Sigma Aldrich) was added for dead cell exclusion, and all stained cells were analyzed in combination with their respective isotype controls using a flow cytometer (Accuri; Ann Arbor, MI USA).

### Reprogramming cells enriched for CD34-expression

The CD34 nucleofection kit and device (Lonza; Allendale, NJ USA) were used for transfections. For CD34+ cells, 3.5 µg of each plasmid in Combination Set 1 and 3 ug of each plasmid for Combination Set 2 except for the L-myc containing plasmid where 2 µg was transfected using program U-08. Cells were seeded onto RetroNectin-coated 6-well plates (Takara Bio, Inc; Otsu, Shiga Japan). Seeding density ranged from 5×10^4^ to 1×10^5^ cells/ml. Reprogramming media: StemSpan SFEM (StemCell Technologies) supplemented with non-essential amino acids (NEAA; Invitrogen), 0.5× Glutamax, N2B27 (Invitrogen), 0.1 mM β-mercaptoethanol (Sigma-Aldrich), 100 ng/mL zebrafish basic fibroblast growth factor (zbFGF), 0.5 µM PD0325901, 3 µM CHIR99021, 0.5 µM A-83-01 (all molecules from Stemgent), and 10 µM HA-100 (Santa Cruz; Santa Cruz CA USA). Conditions for PBMC reprogramming relied on 1×10^6^ cells per transfection, program T-16, and DNA from Combination Set 2 at the concentrations described for CD34+ cells. Reprogramming media for PBMCs was the same with the exception of the small molecule cocktail which contained recombinant human LiF (Millipore), 3 uM CHIR99021, and 0.5 uM A-83-01. In general, cultures were fed with fresh medium every other day for 9 to 14 days then transitioned to TeSR2 (Stem Cell Technologies) without the addition of small molecules. iPSC colonies were scored with Tra-1-81 antibody (StainAlive™ DyLight™ 488 Mouse anti-Human Tra-1-81 antibody; Stemgent) or mouse-anti-Tra-1-60 IgM antibody (R&D) in combination with goat anti-mouse IgM Alexa 488 (Invitrogen), and alkaline phosphatase expression (Vector Blue Alkaline Phosphatase Substrate Kit III, Vector Laboratories; Burlingame, CA USA).

### Detecting endogenous expression of pluripotency markers

Total RNA was isolated using the RNeasy Mini Plus kit (Qiagen; Valencia, CA USA) per the manufacturer's protocol. Approximately 1 µg of total RNA was used for cDNA synthesis using the SuperScript III First-Strand Synthesis system for RT-PCR (Invitrogen). RT-PCR was performed using previously described primers and those listed in Table 6 [2]. cDNA was diluted 1∶2 and 1 µl was used in reactions with GoTaq Green Master Mix (Promega; Madison, WI USA).

**Table 6:** Primer sequences for the detection of endogenous gene expression.

Gene	Size	Primer Name	Primer Sequence (5′ – 3′)
DNMT3B	242	DNMT3B F	TGC TGC TCA CAG GGC CCG ATA CTT C
		DNMT3B R	TCC TTT CGA GCT CAG TGC ACC ACA AAA C
REX1	306	REX1 F	CAG ATC CTA AAC AGC TCG CAG AAT
		REX1 R	GCG TAC GCA AAT TAA AGT CCA GA
TERT	446	hTERT F	CCT GCT CAA GCT GAC TCG ACA CCG TG
		hTERT R	GGA AAA GCT GGC CCT GGG GTG GAG C
UTF1	171	UTF1 F	CCG TCG CTG AAC ACC GCC CTG CTG
		UTF1 R	CGC GCT GCC CAG AAT GAA GCC CAC

Representative sequences for primers that detect gene expression characteristic of pluripotent cells that are endogenously expressed. Size indicates the length of the fragment amplified by each given set of primers. DNMT3, DNA methyltransferase 3; TERT, telomerase reverse transcriptase.

### Episomal and Genomic DNA isolation

Episomal DNA was isolated as previously described and genomic DNA was procured using the QIAamp DNA Blood Mini Kit (Qiagen) according to the manufacturer's protocol [2]. PCR was performed on 140 ng of genomic DNA and 50 ng of episomal DNA using GoTaq Green Master mix. Plasmid DNA was used as a control and diluted to 1 and 0.1 copies per genome.

### Immunoglobulin heavy chain and T cell gene rearrangements

Both T cell gene segment rearrangements as well as immunoglobulin gene (IgH) rearrangements were screened using the T Cell Receptor Gamma Gene Rearrangement and IgH Rearrangement Assay kits for Gel Detection respectively (Invivoscribe; San Diego, CA USA). A subset of iPSCs from either CD34+ cells and PBMCs were screened using the T Cell Receptor Gamma Gene clonality, T cell Receptor Beta Gene Clonality, IgH and IgK B cell clonality Assay kits for ABI fluorescence detection (InvivoScribe).

### Karyotyping

G-banding analysis was performed by Cell Line Genetics (Madison, WI).

### In Vitro Differentiation and Teratoma Studies

iPSC clones from donor 2939 (clones 4 and 5) were differentiated at passage 15 into neurons according to protocols from previously published methods [32], [33]. Cells were fixed with 4% formaldehyde, washed, and permeabilized using 0.1% Triton-X-100 and 1% Normal Donkey Serum in PBS. Cells were incubated with MAP2 (Sigma M1406, 1∶3000) or Tuj1 (Sigma T8660, 1∶5000) antibodies for 2 hours at room temperature, washed, and incubated with A488-conjugated secondary antibodies (Invitrogen, A21121 for MAP2, A21202 for TUJ1). DNA was stained using Hoechst 33342. *Teratomas* - iPSCs grown on MEFs were harvested using collagenase IV (Invitrogen) and injected into hind limb muscles of 6-week old female SCID/beige mice (Harlan Laboratories; Indianapolis, IN USA). Each mouse was injected with one, confluent 6-well plate of cells and a total of 3 mice were injected per cell line. Tumors were embedded in paraffin and sectioned; sections were processed with hematoxylin and eosin staining at the UWCCC Histology Lab at the University of Wisconsin-Madison. Unstained sections were processed with nestin and alpha-feto protein antibodies by the Immunohistochemical Core Service affiliated with the Department of Surgery at the University of Wisconsin-Madison. All animal work was conducted according to relevant national and international guidelines under the approval of the Cellular Dynamics International Animal Care and Use Committee.
